# Ultra-Fast Thermal Shock Evaluation of Ti_2_AlC Ceramic

**DOI:** 10.3390/ma15196877

**Published:** 2022-10-03

**Authors:** Wei Ding, Baotong Hu, Shuai Fu, Detian Wan, Yiwang Bao, Qingguo Feng, Salvatore Grasso, Chunfeng Hu

**Affiliations:** 1 Key Laboratory of Advanced Technologies of Materials, Ministry of Education, School of Materials Science and Engineering, Southwest Jiaotong University, Chengdu 610031, China; 2 State Key Laboratory of Green Building Materials, China Building Materials Academy, Beijing 100000, China

**Keywords:** Ti_2_AlC, ultra-fast thermal shock, microstructure, residual flexural strength

## Abstract

In this work, the rapid thermal shock behavior of Ti_2_AlC ceramics was studied using induction heating. The present evaluation method possesses the merits of very rapid heating within tens of seconds and fast quenching in water of less than 0.1 s, removing the shortcomings of traditional thermal shock. For comparison, the samples were also quenched in the air to investigate the thermal shock mechanisms. The results showed that the abnormal shock occurred in the samples when quenching in water, ascribed to the formed oxide layer on the surface of Ti_2_AlC ceramic inhibited the water penetration into the substrate. The quenched Ti_2_AlC samples still had a high residual flexural strength above 167 MPa up to 1150 °C, exhibiting promising applications in the high-temperature fields.

## 1. Introduction

The term “MAX phases” was first used by Barsoum. Its chemical formula was determined to be M*_n_*_+1_AX*_n_* (*n* = 1–6), where M is the transition group metal element, A is the main group element, and X is carbon, nitrogen, or boron. Generally, MAX phases can be divided into different groups according to the different *n* values, such as 211, 312, and 413 phases [1,2,3,4,5,6,7,8,9]. The studies of MAX phases have shown they combine the excellent properties of metals and ceramics. Like metals, their electrical conductivity, thermal conductivity, and thermal shock resistance are excellent; and like ceramics, MAX phases possess high flexural strength and high Young’s modulus [10,11,12,13,14,15,16,17]. As one of the early MAX phases, Ti_2_AlC ceramic has been studied widely in terms of relatively high strength of more than 400 MPa, relatively low oxidation rate at temperatures up to 1650 °C for a short period of time, good hot corrosion resistance up to 850 °C, excellent damage tolerance, and good chemical stability. It can show the phenomenon of abnormal thermal shock, which is also found in other MAX phase ceramics. Its excellent performance makes it possible to be used in high temperatures and harsh corrosion environments. Therefore, to explore the application possibility in extreme environments, especially at high temperatures, the effective thermal shock behavior of the Ti_2_AlC ceramic should be systematically investigated [18,19,20,21,22,23,24,25].

In previous research, Bai et al. conducted thermal shock tests of Ti_2_AlC ceramic and confirmed that abnormal thermal shock occurred as the temperature increased from 1000 °C to 1400 °C [26]. Similarly, Bao et al. studied the thermal shock behavior of Ti_3_AlC_2_ ceramic by measuring the flexural strength of Ti_3_AlC_2_ samples after water quenching. They determined that the residual flexural strength increased with the increasing temperature between 1000 °C and 1300 °C, also corresponding to the abnormal thermal shock phenomenon [27]. These works have proved the excellent thermal shock resistance of Ti-Al-C MAX phases. However, the testing method has the obvious shortcomings of a slow heating rate, long dwelling time, and slow quenching process, which has a long operating time and could not reflect the service reliability on time.

To address this problem, Su et al. developed a rapid thermal shock performance evaluation method. The Ti_3_SiC_2_ samples were rapidly heated by the energized induction coil within tens of seconds and then fell into the cooling water within 0.35 s. He found no abnormal thermal shock behavior, and the flexural strength continuously decreased with the increasing temperature [28]. Furthermore, Hu et al. studied the rapid thermal shock behavior of Ti_3_AlC_2_ ceramic and confirmed that no abnormal shock behavior existed [29]. Both works pointed out the important positive effect of formed oxide scales on the residual flexural strength.

To evaluate the excellent thermal shock resistance of Ti_2_AlC ceramic before its wide applications, this work focuses on the effect of modified temperature on thermal shock resistance by using the rapid quenching method [28,29]. The samples were rapidly heated in the air and then quenched in water within 0.1 s. The composition, microstructure, and thermal shock mechanism of the quenched Ti_2_AlC ceramic were systematically investigated.

## 2. Experimental Procedures

### 2.1. Sample Preparation

Bulk Ti_2_AlC ceramic was hot-pressed using the lab-made Ti_2_AlC powder, fabricated by the solid–liquid reaction method [19]. The consolidation temperature was 1300 °C and the dwell time was 60 min. The obtained dense bulk had a density of 4.1 g/cm^3^, corresponding to the relative density of 99%. In total, 21 cuboid samples with a dimension of 3 × 4 × 36 mm^3^ were machined by electrical discharged machining (EDM) (DK7732, Ningbo Haishu Zhongyuan Machine Co., Ltd., Ningbo, China) and were chamfered at the edges. All samples were polished down to 1.0 μm diamond grits.

### 2.2. Rapid Thermal Shock Testing

Rapid thermal shock characterization was conducted using a 500–1100 kHz power supply device (Dongguan Jinbenlai Electromechanical Equipment Co., Ltd., Dongguan, China). The detailed description of the lab-made facility can be found in [28,29]. The quenching conditions are listed in Table 1. Based on the experiments, it was found that Ti_2_AlC ceramic decomposed into the TiC phase at the quenching temperature of 1350 °C. Therefore, the quenching temperatures were selected as 480 °C, 920 °C, and 1150 °C, respectively. For each temperature, three samples were tested. The accurate temperature of samples heated by the induction coil was measured by a pyrometer (Raynger 3i Plus, Raytek, Santa Cruz, CA, USA) and recorded on a computer. The water-quenched samples could fall into the water within 0.1 s based on the theory of falling object movement.

### 2.3. Characterization

The flexural strength of quenched samples was tested on a universal testing machine (YC-100KN, Oneyice Corp., Ningbo, China). The span was 30 mm, and the crosshead speed was 0.5 mm/min. The phase characteristics of samples were investigated by an X-ray diffraction (XRD) facility (D8 ADVANCE A25X, Bruker, Karlsruhe, Germany) with Cu *K_α1_* radiation (λ = 0.154 nm, 40 kV and 30 mA) and analyzed by JADE 9 (Materials Data Inc., Newtown Square, PA, USA). The surface morphology and cross-section of the quenched samples were observed by a field emission scanning electron microscope (FSEM) (Apreo 2C, Thermo Scientific, Waltham, MA, USA) equipped with an energy dispersive spectrometer (EDS) for element analysis.

## 3. Results and Discussion

### 3.1. Phase Composition and Microstructure of Air-Quenched Ti_2_AlC Ceramic Heated in Air

**Figure 1:**
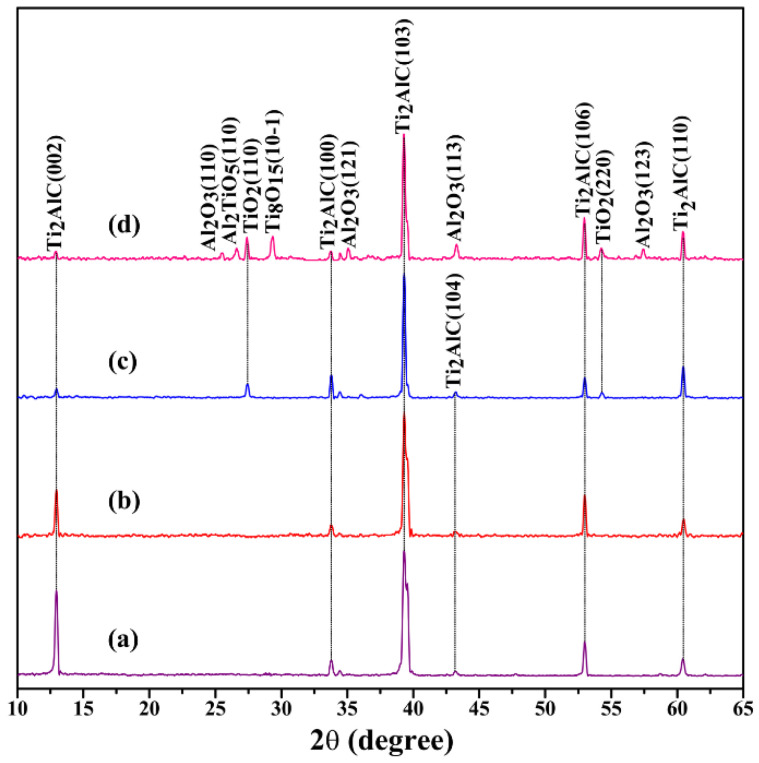
X-ray diffraction (XRD) patterns of Ti_2_AlC samples quenched in air at different temperature: (**a**) 25 °C, (**b**) 480 °C, (**c**) 920 °C, and (**d**) 1150 °C.

Figure 1 displays the X-ray diffraction (XRD) patterns of the Ti_2_AlC samples quenched in air. Table 2 lists the phase compositions formed on the surface of Ti_2_AlC samples at each temperature point. The samples used in this experiment were relatively pure since only the Ti_2_AlC phase could be examined (Figure 1a). When quenching at 480 °C, no other phase could be examined on the sample surface (Figure 1b). While at the quenching temperature of 920 °C, the formed phase of TiO_2_ was detected on the sample surface, which should be ascribed to the reaction between Ti_2_AlC and oxygen (Figure 1c). At the highest quenching temperature of 1150 °C, the newly formed phases of Al_2_O_3_, Al_2_TiO_5_, and Ti_8_O_15_ were examined (Figure 1d). Here, the formation of Al_2_TiO_5_ is due to the reaction between Al_2_O_3_ and TiO_2_. The weak reflections between 45° and 50° are rather small intensities that originate from Ti_2_AlC. The main phase on the sample surface is still Ti_2_AlC, which proves the excellent oxidation resistance of Ti_2_AlC at high temperatures [30,31,32,33].

**Figure 2:**
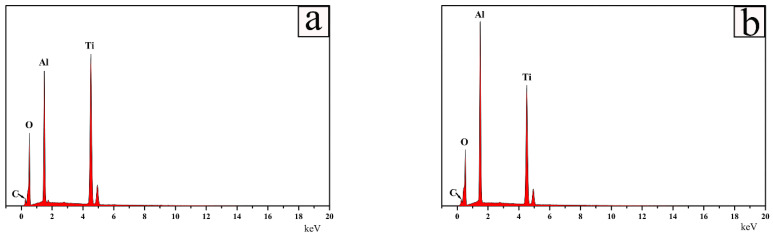
Energy dispersive spectrometer (EDS) patterns of Ti_2_AlC samples quenched in air at different temperatures: (**a**) 920 °C and (**b**) 1150 °C.

To further support the results of the examinations of the XRD patterns, element content on the surface of samples at different quenching temperature using an EDS spot scan were investigated. Figure 2 presents the energy dispersive spectrometer (EDS) patterns of Ti_2_AlC samples quenched in the air at two temperatures. The content of Ti was higher than Al when quenching at 920 °C and demonstrated a larger proportion of TiO_2_ on the surface than Al_2_O_3_, which was examined by XRD (Figure 2a). At the temperature of 1150 °C, Al_2_O_3_ and Al_2_TiO_5_ were more concentrated on the surface than TiO_2,_ which was examined by XRD (Figure 2b), and thus the content of Al was higher than Ti.

**Figure 3:**
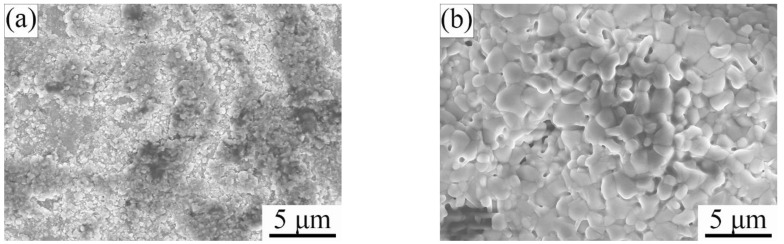
Scanning electron microscope (SEM) micrographs of sample surface of Ti_2_AlC ceramic quenched in air at different temperatures: (**a**) 920 °C and (**b**) 1150 °C.

**Figure 4:**
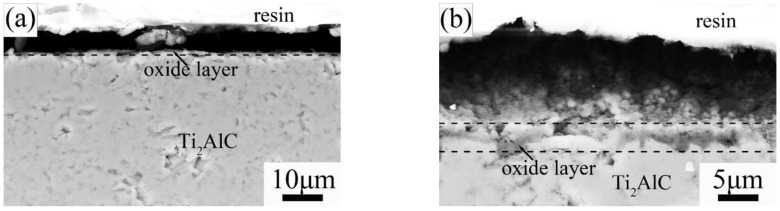
SEM micrographs of sample cross section of Ti_2_AlC ceramic quenched in air at different temperatures: (**a**) 920 °C and (**b**) 1150 °C.

Figure 3 and Figure 4 show the scanning electron microscope (SEM) micrographs of surface and cross-section Ti_2_AlC samples quenched in the air at 920 °C and 1150 °C, respectively. Inhomogeneous and fine oxide particles (less than 1.0 μm) were observed on the quenched surface at 920 °C (Figure 3a). By investigating the cross-section of quenched surface, it is found that no continuous oxide layer exists (Figure 4a). It is confirmed that at 920 °C, the rapid quenching process did not induce the formation of a compact and thick oxide layer. However, when quenched at 1150 °C, it was observed that a large amount of coarse oxide particles (1–2 μm) distributed on the sample surface (Figure 3b) and the formed dense oxide layer with a thickness of 2–3 μm had a strong bonding with the Ti_2_AlC substrate (Figure 4b). After air quenching at 1150 °C, the formed oxide scale seemed to be very dense.

### 3.2. Phase Composition and Microstructure of Water-Quenched Ti_2_AlC Ceramic Heated in Air

**Figure 5:**
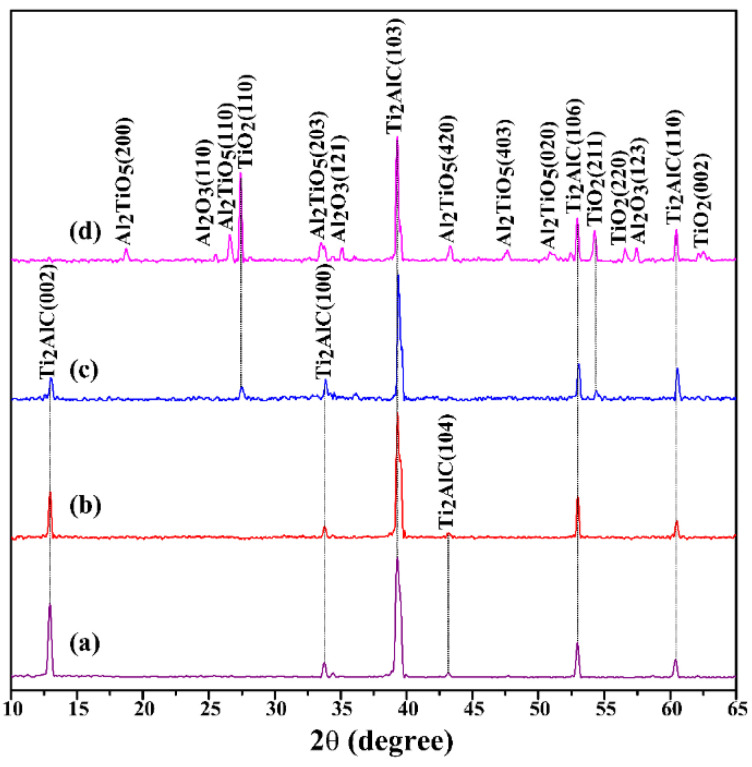
XRD patterns of Ti_2_AlC samples quenched in water at different temperatures: (**a**) 25 °C, (**b**) 480 °C, (**c**) 920 °C, and (**d**) 1150 °C.

**Figure 6:**
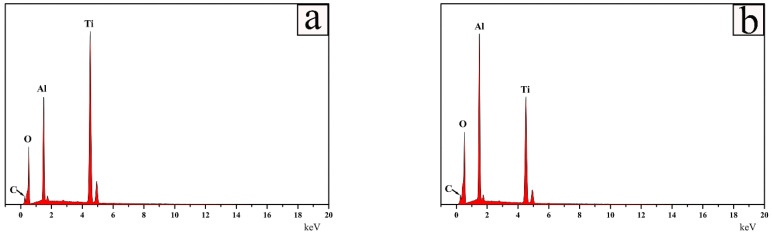
EDS patterns of Ti_2_AlC samples quenched in water at different temperatures: (**a**) 920 °C and (**b**) 1150 °C.

Figure 5 shows the XRD spectrum of quenched Ti_2_AlC samples in water. Table 3 lists the phase compositions of the sample surface of Ti_2_AlC ceramic when quenched in water at different temperatures. Similar to those samples quenched in air, only at the high temperature of 920 °C, there was a formed TiO_2_ phase on the quenched sample surface (Figure 5a–c), exhibiting excellent oxidation resistance. When quenched at 1150 ℃, the phases of TiO_2_, Al_2_O_3_, and Al_2_TiO_5_ were examined on the quenched sample surface (Figure 5d). Here, the appearance of the Al_2_TiO_5_ phase is also attributed to the reaction between TiO_2_ and Al_2_O_3_. Figure 6 displays the energy dispersive spectrometer (EDS) patterns of Ti_2_AlC samples quenched in water at 920 °C and 1150 °C. Similar to those samples quenched in air, the content of Ti was higher than Al when quenching at 920 °C, and the content of Al was higher than Ti at the higher temperature of 1150 °C that support the results of the examinations of TiO_2_ at 920 °C and Al_2_O_3_ and Al_2_TiO_5_ at 1150 °C.

**Figure 7:**
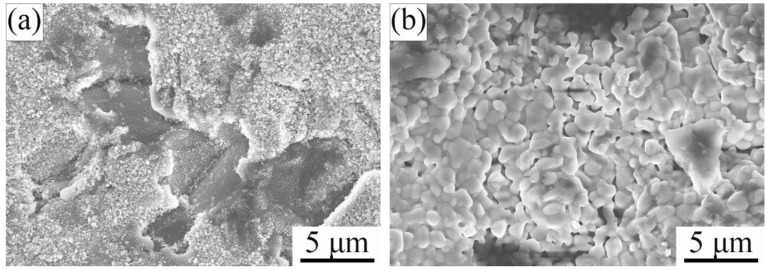
SEM micrographs of sample surface of Ti_2_AlC ceramic quenched in water at different temperatures: (**a**) 920 °C and (**b**) 1150 °C.

**Figure 8:**
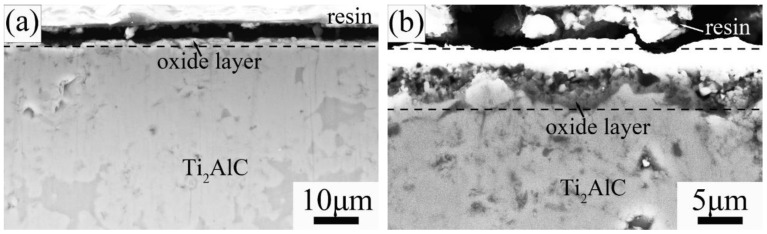
SEM micrographs of sample cross-section of Ti_2_AlC ceramic quenched in water at different temperatures: (**a**) 920 °C and (**b**) 1150 °C.

Figure 7 and Figure 8 show the SEM micrographs of the surface and cross-section of the Ti_2_AlC samples quenched in water, respectively. On the quenched sample surface at 920 °C, only the very fine TiO_2_ particles less than 1 μm were observed and did not cover the whole surface, indicating the invalid protection during the quenching process (Figure 7a and Figure 8a). When quenched at 1150 °C, the coarse particles with the size of 1–3 μm cover the whole quenched sample surface (Figure 7b), and the thickness of the oxide layer is about 5 μm (Figure 8b). By comparing the Ti_2_AlC sample surface quenched at 1150 °C in air and water, it was observed that the oxide layer of Ti_2_AlC ceramic quenched in water was separated into two layers: the top layer is very loose, and the subjacent layer is dense to bonding the substrate. It was concluded that the water penetrated the oxide layer during the water quenching but did not reach the Ti_2_AlC matrix.

### 3.3. Residual Flexural Strength of Ti_2_AlC Ceramic

**Figure 9:**
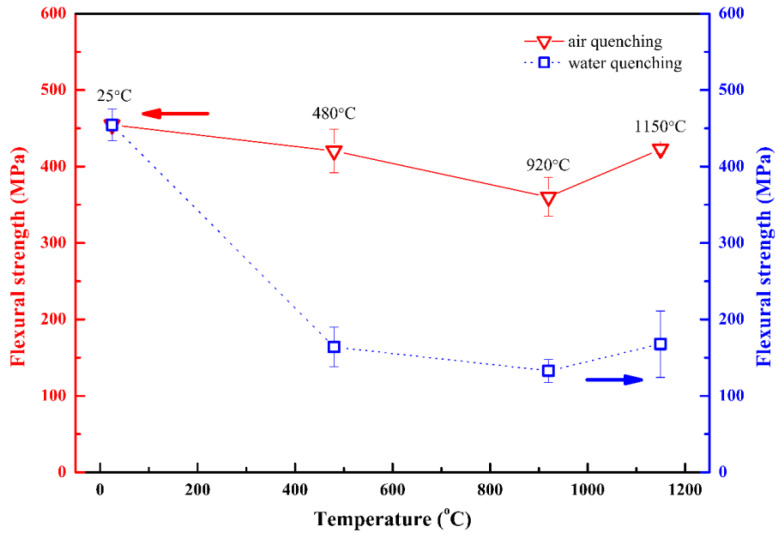
Residual flexural strength of air-quenched and water-quenched Ti_2_AlC samples rapidly heated in air.

Figure 9 shows the flexural strength of Ti_2_AlC samples after quenching in air and water. It is seen that below 920 °C, the residual flexural strength continuously decreases with the increment of quenching temperature. The decreasing tendency of the strength of samples quenched in the air has a slower change in comparison with that quenched in water. At the temperatures of 25 °C, 480 °C, and 920 °C, the residual flexural strengths of samples are 454 MPa, 420 MPa, and 360 MPa, respectively, when quenched in air, and those are 454 MPa, 164 MPa, and 132 MPa, respectively, when quenched in water. Interestingly, when the quenching temperature was increased up to 1150 °C, the residual strength of samples showed an abnormal enhancement up to 422 MPa and 167 MPa, respectively, in air and in water. In comparison with the previous rapid thermal shock tests, the abnormal thermal shock behavior did not occur in the quenched Ti_3_SiC_2_ and Ti_3_AlC_2_ ceramics [28,29], which proves the more excellent thermal shock resistance of Ti_2_AlC ceramic.

### 3.4. Thermal Shock Mechanism of Ti_2_AlC Ceramic

During the quenching, the rapid temperature degradation plays a key role in affecting the residual flexural strength by inducing large tensile stresses. The tensile stresses of samples were calculated according to the Equation [34]: $\sigma =\frac{\alpha E∆T}{1-\nu }$, where α is coefficient of thermal expansion, E is elastic modulus, ∆T is temperature difference during quenching, and ν is Poisson’s ratio. For the Ti_2_AlC samples, the values of these parameters are as follows: E = 278 GPa, ν = 0.25, α = 9 × 10^−6^ K^−1^, respectively [31,32,35,36]. The temperature of the cooling medium is 25 °C, and the temperatures of quenching are 480 °C, 920 °C, and 1150 °C, respectively. Thus, the calculated transient tensile stresses are 1.5 GPa, 2.9 GPa, and 3.7 GPa, respectively.

Based on the SEM micrographs in Figure 3, Figure 4, Figure 7 and Figure 8, it was seen that below 920 °C, there were no homogeneous oxide layers on the quenched surface of Ti_2_AlC samples. Therefore, the significant tensile stresses directly impacted the sample surface to degrade the flexural strength [26,27,28]. However, when the temperature was increased to 1150 °C, the thick oxide layers formed on the sample surfaces when quenching in water and air, which could effectively enlarge the thermal gradience as the thermal barrier to weaken the effect of tensile stresses. When quenched in water, the water also penetrated the supernal oxide layer (Figure 8b). Fortunately, the subjacent dense oxide layer adhering to the Ti_2_AlC matrix was not damaged, which could still protect the substrate. As a result, the residual flexural strength of quenched Ti_2_AlC samples exhibited an abnormal increase, presenting excellent thermal shock resistance.

## 4. Conclusions

The ultra-fast thermal shock behavior and mechanism of Ti_2_AlC samples were investigated by quenching in water and air. The obtained results are summarized as follows:

(1) Below 920 °C, no homogeneous oxide layer formed on the quenched surface of Ti_2_AlC ceramic. Above 1150 °C, the thick oxide layers covered both the quenched surfaces of Ti_2_AlC samples in water and air.

(2) Below 920 °C, the residual flexural strength of Ti_2_AlC ceramic continuously decreased incrementally with the quenching temperature. When quenched in air, the residual flexural strength decreased from 454 MPa to 360 MPa. Those samples quenched in water showed a decrease from 454 MPa to 132 MPa. Interestingly, when quenched at 1150 °C in water and air, the residual flexural strength of samples was abnormally increased to 422 MPa and 167 MPa, respectively.

(3) The continuous decrease of residual flexural strength was ascribed to the significant tensile stresses generated on the quenched surface of the Ti_2_AlC ceramic, which was not protected by the homogeneous oxide scale. The abnormal increase in residual flexural strength was associated with the thermal barrier effect of a thick oxide layer, which could effectively weaken the negative effect of thermal stresses.

(4) The results indicate that the Ti_2_AlC ceramic possesses excellent thermal shock resistance above 1150 °C and could find potential use in high-temperature fields.

## Figures and Tables

**Table 1:** Conditions of rapid thermal shock testing of Ti_2_AlC ceramic.

Divisions	Heating	Quenching
Group 1	In air	In air
Group 2	In air	In water

**Table 2:** Phase compositions of sample surface of Ti_2_AlC ceramic quenched in air at different temperatures.

Temperature	Phase Compositions
25 °C	Ti_2_AlC
480 °C	Ti_2_AlC
920 °C	Ti_2_AlC, TiO_2_
1150 °C	Ti_2_AlC, TiO_2_, Al_2_O_3_, Al_2_TiO_5_, Ti_8_O_15_

**Table 3:** Phase compositions of the sample’s surface of water-quenched Ti_2_AlC ceramic heated in air at different temperatures.

Temperature	Phase Compositions
25 °C	Ti_2_AlC
480 °C	Ti_2_AlC
920 °C	Ti_2_AlC, TiO_2_
1150 °C	Ti_2_AlC, TiO_2_, Al_2_O_3_, Al_2_TiO_5_

## Data Availability

All the data is available within the manuscript.
